# Performance Evaluation of the Sizing of Cotton Warp Yarns Using Low-Cost Carboxymethyl Cellulose Derived from Saudi Wheat Straw

**DOI:** 10.3390/polym18020226

**Published:** 2026-01-15

**Authors:** Samah Maatoug, Elham Abu Nab

**Affiliations:** Department of Fashion and Textiles Design, Faculty of Art and Design, University of Tabuk, Tabuk 71491, Saudi Arabia; eabunab@ut.edu.sa

**Keywords:** warp sizing, wheat straw, carboxymethyl cellulose, biodegradability, adhesive and cohesive force, desizing efficiency, cost-effectiveness

## Abstract

Sizing is a critical operation in woven fabric production, as it enhances weaving efficiency by improving warp yarn performance. Conventional sizing agents include maize starch, polyvinyl alcohol (PVA), and commercial carboxymethyl cellulose (CMC). In this study, a low-cost and biodegradable carboxymethyl cellulose derived from wheat straw (CMC_ws_) was investigated as an alternative sizing agent for cotton open-end yarns with a count of Nm 12.2. The high degree of substitution (*DS* = 1.23) of CMC_ws_ indicates extensive carboxymethylation, which enhances the polymer’s hydrophilicity and solubility in water. This, in turn, contributes to a higher apparent viscosity (*η* = 903.03 cP at 300 s^−1^), reflecting stronger molecular chain interactions and better film-forming ability. CMC_ws_ was applied using a high-pressure squeezing technique, and its effect on yarn performance was evaluated in terms of tensile properties, film characteristics, and yarn surface morphology. The results showed that CMC_ws_ provided a tenacity gain of 28.57%, a hairiness reduction of 54.34%, and an abrasion resistance gain of 37.14%. These values fall within acceptable industrial ranges and are comparable to those obtained using conventional sizing agents. Furthermore, the optimized CMC_ws_ formulation, containing plasticizer and lubricant additives, exhibited good desizing efficiency, with effective removal achieved in hot water. The findings indicate that wheat-straw-derived CMC_ws_ is a viable, sustainable alternative to traditional sizing agents for woven fabric production.

## 1. Introduction

During weaving, warp yarns undergo continuous mechanical and physical loads, including bending, dynamic tension, friction, and static electricity [1,2]. These stresses often compromise the integrity of the yarns, leading to breakages and decreased weaving efficiency. Sizing is a critical process that addresses these challenges by coating or impregnating the yarns with a polymer, thereby enhancing their mechanical performance and weavability [3,4,5]. Among various lignocellulosic feedstocks, wheat straw is particularly attractive due to its widespread availability and underutilization as an agricultural residue.

In regions such as Saudi Arabia, wheat production reaches approximately 700,000 tons annually [6]. Based on the findings of Pan and Sano [7], harvesting wheat grain yields a substantial quantity of straw, with production ratios averaging between 1.3 and 1.4 kg of straw per kilogram of grain. This indicates a substantial biomass resource that remains largely untapped. Wheat straw typically contains cellulose in the range of 34.6% to 41.4%, with holocellulose content reported to be around 72% [8,9], thereby establishing its suitability as a feedstock for biopolymer synthesis and other value-added applications.

This study investigates the use of carboxymethyl cellulose (CMC_ws_) derived from Saudi wheat straw as a low-cost, biodegradable sizing agent for cotton warp yarns. CMC_ws_, a water-soluble derivative of cellulose, is already employed in various industries, including textiles, due to its excellent film-forming and adhesive properties.

The research emphasizes both the environmental and economic advantages of using wheat straw-derived CMC_ws_ and evaluates its performance in textile applications. A comparative analysis was conducted to assess the weavability of cotton warps sized with CMC_ws_ from wheat straw against conventional sizing agents, including maize starch, polyvinyl alcohol (PVA), and industrial-grade commercial CMC. The evaluation focused on key yarn performance indicators such as breaking strength, elongation at break, abrasion resistance, and overall weaving efficiency.

By leveraging an abundant and sustainable resource like wheat straw, this study aims to contribute to the development of eco-friendly textile processing solutions while meeting the technical demands of modern weaving processes.

## 2. Materials and Methods

### 2.1. Materials and Reagents

Wheat straw, an agricultural byproduct, was sourced from a local farm in Tabuk, located in Saudi Arabia. The collected straw was initially cut into small pieces and subsequently dried in a convection oven at 50 °C for 24 h to reduce moisture content. After drying, the straw was ground using a kitchen blender to produce a fine powder. The powdered material was then sieved, and only particles passing through a 60-mesh sieve were retained for further processing.

All reagents and chemicals utilized in this study, including those required for the synthesis and characterization of carboxymethyl cellulose (CMC_ws_), were obtained from Sigma-Aldrich (St. Louis, MO, USA) and used without further purification (Sodium hydroxide (≥98%), monochloroacetic acid (99%), 1-butanol (≥99.5%), and glycerol (≥98%)). Commercial CARBO 150 RH (CMC) and PVA 2488, obtained from a local supplier (SITEX, Ksar Hellal, Tunisia), were employed as reference materials to evaluate the performance of the synthesized CMC_ws_. Distilled water was used for all preparations.

### 2.2. Chemical Composition

The standard Technical Association of the Pulp and Paper Industry (TAPPI) methods were used to determine the chemical composition of the Saudi wheat straw for various components, including ash (T211 om-93), α-cellulose (T203 cm-99), 1% sodium hydroxide solution solubility (T212 om-12), ethanol–toluene solubility (T204 cm-07), cold and hot water solubility (T207 cm-08), and Klason lignin (T222 om-88). In each case, the average of three tested samples was reported.

### 2.3. Isolation of Cellulose from Wheat Straw

The acid-chlorite method was used to separate holocellulose from Saudi wheat straw [9,10]. In order to remove water-soluble materials, 10 g of dried and sieved Saudi wheat straw was first steeped in 200 mL of distilled water at 75 °C for two hours. Following this processing, the material was delignified using a 1.7% sodium chlorite solution that was kept at a pH of 3.5 to 4.0 by adding 6 M acetic acid. After keeping the mixture at 75 °C for two hours, the delignification cycle was repeated three or four times until the sample turned completely colorless, indicating that the lignin had been successfully removed.

Holocellulose was treated with a 10% potassium hydroxide solution at room temperature for 16 h in order to solubilize the hemicellulose fraction and separate cellulose. The remaining cellulose was then thoroughly washed with distilled water until the filtration became neutral. The purified cellulose was dried at ambient temperature prior to further use.

### 2.4. Cellulosic Fractions’ Carboxymethylation

**Figure 1:**
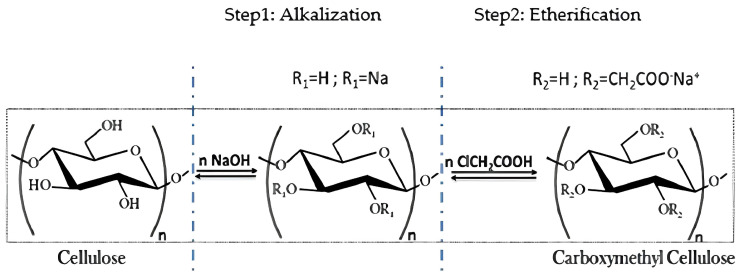
Schematic representation of synthetic route of carboxymethylaion of cellulose.

The transformation of Saudi wheat straw isolated cellulose into carboxymethyl cellulose (CMC_ws_) was carried out in two main steps—alkalization followed by etherification—as illustrated in Figure 1. The carboxymethylation procedure was adapted from [11] with slight modifications.

For the alkalization step, 10 g of purified cellulose was mixed with 60 mL of *n*-butanol and 60 mL of 40% aqueous sodium hydroxide solution. The mixture was stirred continuously at 60 °C for 12 h to convert the hydroxyl groups (–OH) of the cellulose into alcoholate groups (–ONa), thereby activating the cellulose for subsequent etherification [12,13].

Following alkalization, the etherification reaction was initiated by adding the required amount of monochloroacetic acid MCA (ClCH_2_COOH) to the reaction mixture. The mixture was then maintained at 60 °C for an additional 6 h to facilitate the substitution of hydroxyl groups with carboxymethyl groups.

Upon completion of the reaction, the slurry was neutralized using glacial acetic acid until the pH reached the range of 6 to 8. The crude CMC_ws_ product was precipitated by suspending the reaction mixture in 800 mL of ethanol. To remove residual salts and byproducts, the CMC_ws_ was washed five times with a 70% ethanol solution. To achieve a state of pure CMC_ws_, the sample was then dried in an oven at 50 °C for 24 h until a consistent weight was attained and stored in polyethylene bags [9,10,14].

The percentage yield of the CMC_ws_ was calculated based on the dry weight of the purified product relative to the initial cellulose mass, and it reaches 90%.

To evaluate the degree of substitution (*DS*) of the prepared CMC_ws_, 5 g of the sample was introduced into 200 mL of a nitric acid–methanol mixture (HNO_3_–CH_3_OH, 1:1 *v*/*v*). The suspension was agitated and allowed to stand for 3 h. Excess acid was subsequently removed by washing the material with 70% methanol until a neutral pH was reached. After drying, 2 g of the sample was dissolved in 200 mL of distilled water, followed by the addition of 30 mL of 1 N NaOH. The resulting solution was then titrated using 1 N HCl. The *DS* value of the CMC was calculated according to the following Equations (1) and (2) [9,12,13]:

(1) $$DS=\frac{0.162A}{1-0.058A}$$

(2) $$A=\frac{\left(BC\right)-\left(DE\right)}{F}$$

where *A* is the equivalent weight of alkali needed per gram of sample, *B* is the amount of NaOH solution (mL), *C* is the NaOH solution’s normality (N), *D* is the amount of HCl solution (mL), *E* is the HCl solution’s normality (N), and *F* is the sample weight (g).

Rheological analyses were performed to determine the apparent viscosity (*η*) of the synthesized CMC_ws_. Measurements were conducted using a controlled-speed rotational rheometer (RC30 Rheotec, Rheotec Messtechnik GmbH, Dresden, Germany) equipped with a cone and plate geometry.

The average degree of polymerization (*DP*) of the biomass corresponds to the mean number of anhydroglucose units (AGU, 162.1 g·mol^−1^) forming the polymer chains and does not account for molecular weight distribution. The DP value can be significantly influenced by the biomass source and the extraction process.

In this study, the average viscosimetric degree of polymerization (*DP_v_*) of the wheat straw extracted polymer was determined by a viscosimetric method using cupriethylenediamine (CED) as solvent, following AFNOR standard NF G 06-037 [15]. The *DP_v_* values were calculated from intrinsic viscosity (*η*) measurements using the Mark–Houwink equation:

(3) $$[\eta ]=K\times DP_{v}^{\alpha }$$

where *K* = 7.5 × 10^−3^ dL·g^−1^ and *α* = 1 at 25 °C.

The corresponding average viscosimetric molar mass (*M_v_*) was calculated as *M_v_ = DP_v_ × 162*.

### 2.5. Sizing Formula

In this study, the research focused on evaluating several sizing agents, including maize starch, polyvinyl alcohol (PVA), commercial carboxymethyl cellulose (CMC), and carboxymethyl cellulose derived from wheat straw (CMC_ws_). These sizing agents were applied to cotton warps to assess their performance in weaving.

As presented in Table 1, each size formulation included not only the primary sizing agent at variable concentration (from 0.5% to 8%) but also a plasticizer agent content (glycerol, 8%) and a lubricant agent content (Avirol, 7%). Glycerol was used to enhance the flexibility and workability of the sizing, while Avirol, a commercial lubricant made of fatty acids, fatty alcohols, and emulsifiers, was included to reduce friction between the yarns and other parts of the weaving machinery. The inclusion of these additives aimed to improve the overall performance of the sized cotton yarns during the weaving process [16].

The behavior of CMC_ws_ under varying pH conditions is strongly influenced by the ionizable carboxymethyl groups along its polymer chain. In neutral to mildly alkaline environments, these groups remain deprotonated, maintaining good solubility, viscosity, and stable film formation, which is favorable for sizing performance. Under acidic conditions, protonation of the carboxylate groups reduces solubility and may lead to aggregation or gel formation, potentially affecting film integrity and desizing efficiency. Conversely, strongly alkaline conditions can induce partial hydrolysis of polymer chains, decreasing viscosity and weakening the cohesion of the sizing film. These pH-dependent properties highlight the importance of maintaining suitable processing conditions to ensure optimal CMC_ws_ film performance, efficient desizing, and protection of cotton yarns during weaving [17].

### 2.6. Preparation of Size Film

The sizing agent was first dissolved in distilled water. As the temperature of the reaction vessel reached 65 °C, the size granules were gradually introduced under continuous stirring. The addition of granules was halted once the temperature approached 90 °C, at which point the heat supply was turned off while stirring was maintained. After the sizing agent formed a uniform solution, the required amounts of lubricant and plasticizer were added and mixed for an additional 30 min. Approximately 10 mL of the prepared sizing formulation was then poured into Petri dishes and allowed to dry under ambient conditions.

### 2.7. Sizing Procedure

Cotton open-end warp yarns with a count of Nm 12.2, 470 rpm twist, supplied by SITEX (Ksar Helal, Tunisia), were investigated in this experiment. The yarns were sized in parallel ends using a laboratory apparatus simulating an industrial sizing machine, operating at a constant speed of 2–3 m/min. The system included an immersing roller and two adjustable squeezing rollers applying a pressure of 220–330 N/m, with the sizing solution maintained at ~90 °C using a built-in thermostat. Following immersion and squeezing, the yarns were dried in a controlled heating chamber to ensure uniform moisture removal and subsequently conditioned under standard atmospheric conditions (65 ± 2% RH, 20 ± 2 °C) for 48 h prior to testing. Using the following formula, the weights of the oven-dried sized and unsized yarn samples that were about 10 m long (three readings per sample) were used to determine the add-on (%).

(4) $$Size\;Add-on\;\left(\%\right)=\frac{weight\;of\;sized\;yarn-weight\;of\;unsized\;yarn}{weight\;of\;unsized\;yarn}\times 100$$

### 2.8. Weavability Evaluation of CMC_ws_-Sized Warp Yarns

The intrinsic qualities of the yarn, the features of the sizing material used, and the resulting attributes of the sized yarn itself all have an impact on the weaving performance of any sized warp yarn [5]. These parameters can be systematically quantified to assess the specific influence of sizing agents on yarn weavability. Understanding this contribution is crucial, as it can be directly correlated with key metrics affecting weaving performance, particularly in relation to yarn failure mechanisms during the weaving process.

In light of this, various commercial sizing agents were selected and characterized with respect to their influence on weavability. These sizes were subsequently applied to warp yarns, which were then exposed to simulated weaving conditions in the laboratory. Through this approach, the weavability of the sized yarns was evaluated under controlled mechanical stress to replicate real-world weaving environments. The following parameters were measured in order to evaluate the weavability of CMC_ws_-sized warp yarns:

- Adhesive force: The adhesive capacity of the sizing formulation was evaluated through its ability to bond the constituent fibers within the yarn structure. Since unsized yarn consists of loosely associated fibers with limited cohesion, the enhancement of yarn tensile strength after sizing serves as an indicator of the adhesive efficiency of the size on the fiber substrate. The breaking strength and elongation at break of the sized yarns were determined using a Textechno Lloyd LR5K dynamometer (Textechno H. Stein GmbH & Co. KG, Mönchengladbach, Germany), following the procedure specified in ISO 2062 [2,18,19,20]. A preliminary yarn tension of 0.5 cN/tex was applied, and each test was conducted such that rupture occurred within 20 ± 3 s. For statistical reliability, 100 measurements were performed for each yarn sample.
- Cohesive force: The cohesion power of sizing materials was assessed by determining the tensile properties of the corresponding size films. Approximately 10 mL of each prepared sizing solution was cast into Petri dishes and allowed to dry under ambient laboratory conditions. Using a Lloyd LR5K tensile tester, the tensile characteristics of size films were assessed in accordance with ASTM standard D882 [20,21,22,23]. Twenty film strips (10mm × 100mm) for each recipe of size solution were tested. Cohesion power (*Coh*, *MPa*) was calculated by dividing the maximum load (N) by the cross-sectional area (m^2^) as follows:
  (5) $$Coh\;\left(MPa\right)=\frac{P}{b\times d}$$
  In this equation, *P* represents the maximum load (N), *b* denotes the sample width (mm), and *d* corresponds to the film thickness (mm). The thickness of each film was measured at five different locations, and the average value was recorded using a digital micrometer with an accuracy of 0.01 mm.
- Superficial structure of the yarn: The fibrous arrangement in cotton warp yarns is not perfectly organized and presents various defects, such as fiber ends, hooks, loops, gimlets, buttons, and others. Sizes are applied to spun warp yarns to reduce their hairiness Index, thereby minimizing the tendency for adjacent yarns to entangle [22,24,25,26]. To evaluate the yarn hairiness before and after sizing, we used a Shirley Electronic Yarn Hairiness Tester against the standard test method ASTM D5647 [20]. For each measurement, 50 m of yarn was tested at a travel speed of 30 m/min. To compare the yarn hairiness and unevenness appearance of untreated and sized yarns, we utilized a Scanning Electron Microscopy (SEM).
- Abrasion resistance: Fabric samples measuring 20 cm × 20 cm were coated with the respective sizing formulations and dried under control conditions (50 samples for each sizing agent). The dried specimens were mounted on a vertical abrasion tester (Branca Idealair 66, Branca S.r.l., Milan, Italy) and subjected to continuous rotational rubbing against 400-mesh emery paper under a constant pressure of 50 g·cm^−2^, following conditioning at 20 ± 2 °C and 65 ± 2% relative humidity for 48 h. Abrasion was continued until the formation of the first visible hole, and the number of abrasion cycles required was recorded as the measure of abrasion resistance.

### 2.9. Desizing Evaluation

The sizing solution was applied to 100% cotton fabrics by immersion under controlled laboratory conditions. After impregnation, the fabrics were dried to a constant weight and subsequently subjected to desizing treatments in a water bath under specific bath ratio, temperature, and time conditions. Complete desizing was achieved by boiling the fabrics in water at a bath ratio of 1:50 (kg fabric: L water) for 30 min, until a constant weight was obtained. To assess desizing performance, the sized fabrics, characterized by known size add-on values (%), were rinsed in water at 90 °C for three successive cycles, each lasting 5 min, using a bath ratio of 1:10 (kg fabric: L water). After rinsing, all samples were oven-dried at 105 °C for 4 h, and their weights were measured to determine the amount of size removed. The desizing efficiency *DE (%)* was obtained based on mass differences by the following Equation (6) [27]:

(6) $$DE\;\left(\%\right)=\;\frac{mass\;of\;rinced\;fabric\;\left(g\right)-mass\;of\;desized\;fabric\;\left(g\right)}{mass\;of\;desized\;fabric\;\left(g\right)\times \;size\;add-on\;\left(\%\right)on\;the\;fabric}\times 100$$

### 2.10. Biodegradation Performance

The biodegradability of Chemical Oxygen Demand (COD) and Biochemical Oxygen Demand (BOD_5_) of CMC_ws_ size was determined. As shown in Table 2, COD was determined according to the GB 11914-89 standard [21,27]. Briefly, a known volume of the sample was mixed with a strong oxidizing agent (potassium dichromate) in acidic conditions and digested at 148 °C for 2 h in a sealed reflux apparatus. After cooling, the remaining dichromate was titrated with ferrous ammonium sulfate, and COD was calculated as the equivalent oxygen consumed (mg O_2_/L). COD measurements were performed in triplicate for each sizing agent to ensure reproducibility. BOD_5_ was measured following the HJ 505-2009 standard [21,27]. For each sample, three replicates were incubated in BOD bottles at 20 ± 1 °C under aerobic conditions for 5 days. The dissolved oxygen (DO) concentration was recorded at the beginning and end of the incubation period using a calibrated DO meter. The BOD_5_ value was calculated as the difference between initial and final DO, corrected for dilution and blanks, and expressed in mg O_2_/L.

The BOD_5_/COD ratio was then used as the biodegradability indicator, with ratios >0.4 indicating readily biodegradable materials and <0.3 indicating poorly biodegradable materials.

## 3. Results and Discussion

### 3.1. Characterization of Extracted Cellulose

The chemical composition of Saudi durum wheat straw, used as an agricultural by-product, was determined and presented in Table 3.

The results indicate that the studied material contains a high α-cellulose content (41 g per 100 g of dry material) and a relatively low lignin content (14 g per 100 g of dry material). This characteristic is particularly important for assessing which is essential for evaluating its suitability as a feedstock for carboxymethyl cellulose (CMC_ws_) production.

The purity of CMC_ws_ (%) was determined as the ratio of the final dried weight to the initial biomass weight, yielding a purity of approximately 99%, indicating a high degree of conversion and minimal residual impurities.

The degree of substitution (DS) of the synthesized carboxymethyl cellulose (CMC_ws_) attained a value of 1.23 at monochloroacetic acid (MCA) concentration of 25 g per 10 g of dry cellulose, indicating a high level of carboxymethylation and hydrophilic character.

Correspondingly, the apparent viscosity (η) measured at room temperature and a shear rate of 300 s^−1^ increased to 903.03 cP, demonstrating the enhanced chain substitution and hydrophilicity of the resulting polymer.

The extracted cellulose exhibited a DP_v_ of approximately 933, corresponding to a viscosimetric molar mass of 151,200 g·mol^−1^.

### 3.2. Adhesive Force of CMC_ws_-Sized Warp Yarns

#### 3.2.1. Effect of Size Concentration

Sizing materials and cotton fiber interaction were assessed by measuring the tensile strength of the sized cotton warp. This parameter reflects the degree of adhesion between the size film and the fiber surface, which directly influences the yarn’s performance during weaving.

Four different sizing agents, maize starch, carboxymethyl cellulose (CMC), polyvinyl alcohol (PVA), and wheat straw-derived carboxymethyl cellulose (CMC_ws_), were applied at varying concentrations, and their effects on the mechanical properties of the cotton yarn were summarized in Table 4.

Table 4 shows yarn breaking strength (cN/Tex) and corresponding breaking strength gain (%) across increasing size concentrations (0.5–8%) for different agents. Breaking strength rose steadily with higher concentrations, indicating improved fiber cohesion and load transfer. CV% values highlight the relative consistency of each agent’s effect.

**Figure 2:**
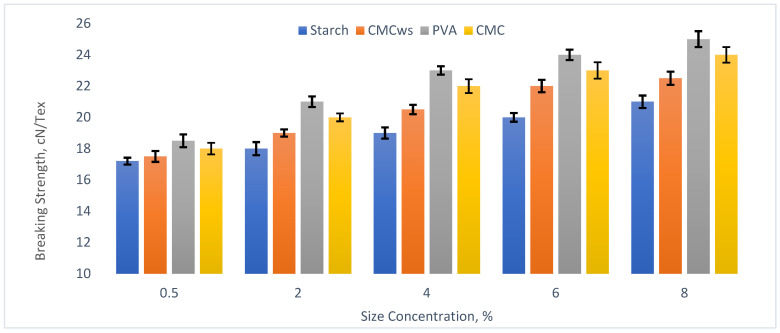
Effect of size concentration on breaking strength of CMC_ws_-sized yarns.

The sizing process involves several key stages: wetting, spreading, and film formation on the fiber surface. The size material retained between the fibers helps to bind the fibers together, while the sizing film on the yarn surface provides protection. As shown in Figure 2, the breaking strength increases with the rising sizing concentration.

As the CMC_ws_ concentration increases, a greater number of sizing molecules engage in covering the fiber surface and bonding the fibers together. The thickness of the CMC_ws_ film deposited on the yarn surface also increases, thereby enhancing the contribution of the sizing film strength to overall yarn–sizing adhesion. When the concentration reaches 0.5%, the breaking strength of warps sized with different agents shows significantly higher values.

Table 5 presents linear regression results obtained using Minitab^®^ 22 at a 95% confidence level. This analysis indicates that the adhesion of the CMC_ws_ sizing material to the fiber is influenced by the sizing concentration. Both the strength of the sizing film and the inherent strength of the fibers contribute to the adhesion force measured using the yarn method, particularly when the sizing concentration exceeds 0.5%.

Regression analysis was performed at a 5% significance level, and the results (*p* < 0.05) demonstrate a statistically significant effect of sizing concentration on yarn breaking strength (cN/Tex). For CMC_ws_, the high coefficient of determination (R^2^ = 95.68%) indicates that the regression model explains most of the variability in the response, confirming its strong predictive accuracy.

#### 3.2.2. Influence of Size Squeeze Pressure

The breaking strengths of yarns sized with various materials, starch, PVA, CMC, and CMC_ws_, are presented in Table 6.

It can be observed that the breaking strength of the yarn increases after sizing, compared to the unsized yarn. However, this improvement in strength is accompanied by a reduction in breaking extension. Notably, the application of high-pressure squeezing during the sizing process results in an increase in the breaking strength of yarns treated with the CMC_ws_ sizing agent. In terms of uniformity, the breaking strength coefficient of variation (CV) for CMC_ws_ was 12.46% at low pressure and 17.28% at high pressure, reflecting relatively consistent yarn properties compared to other sizing agents.

It is evident that yarns sized under high pressure consistently demonstrate improved weaving performance compared to those sized under low pressure. This observation was further supported through structural analysis of the yarn. The increased binding force achieved through sizing enhances the cohesion between fibers, promoting a more uniform roller load distribution during deformation. As a result, the yarn exhibits higher breaking strength and reduced extensibility.

Yarn packing density, defined as the ratio of the cross-sectional area of the fibers to that of the yarn, was measured using an optical microscope. Packing density values range from 0 to 1, where 0 represents completely loose fibers with no compaction, and 1 represents maximum compaction with fibers fully occupying the yarn cross-section.

As shown in Table 7, yarn packing density increases after sizing, with the highest values observed under high squeeze pressure, indicating that increased pressure compresses the yarn structure and enhances its compactness. While low squeeze pressure leads to a slight increase in yarn diameter compared to unsized yarn, high-pressure squeezing at the same size add-on yields diameters comparable to or smaller than those of unsized yarn. These results confirm that squeeze pressure during sizing compacts the yarn, with compactness rising proportionally to pressure. Reduced yarn diameter is advantageous for weaving, facilitating smoother passage through the reed dents.

Higher packing density enhances fiber cohesion and minimizes interfiber slippage, thereby improving weaving performance [2]. Our structural analysis indicates that the superior weaving performance of CMC_ws_-sized yarns under high-pressure squeezing results from uniform size coating, deeper size penetration, and increased packing density.

Size penetration serves a dual function: it strengthens interfiber binding and provides a stable base for the surface size coating. While the coating protects the yarn and encapsulates protruding fibers, excessive penetration and coating can be detrimental, causing greater size shedding during weaving. In yarns sized under high pressure, although the film thickness is lower, size penetration is greater than in low-pressure-sized yarns with the same size add-on. This enhanced penetration improves interfiber binding and secures the size film to the yarn surface, thereby increasing abrasion resistance [5].

### 3.3. Cohesive Force of CMC_ws_-Sized Warp Yarns

The cohesive force of sizing materials plays a critical role in their ability to protect the yarn surface during the weaving process. This property is commonly assessed through the film strength, which reflects the internal cohesion of the size material and its capacity to form a durable, protective layer around the yarn. In this study, the breaking strengths of size films prepared from various materials including starch, polyvinyl alcohol (PVA), commercial carboxymethyl cellulose (CMC), and wheat-straw-derived CMC (CMC_ws_) were evaluated and summarized in Table 8. Among these, PVA exhibited the highest cohesive force, indicating its superior film-forming ability.

Nevertheless, CMC_ws_ also demonstrated considerable film strength, suggesting its effectiveness in forming a cohesive and protective coating on the yarn surface. This performance implies that CMC_ws_ can contribute to improved weavability by enhancing fiber cohesion and reducing yarn hairiness and breakage during weaving. It can be inferred that a CMC_ws_-based sizing agent with high cohesive strength and strong adhesion to the fiber substrate is likely to offer enhanced weavability and weaving efficiency [18].

### 3.4. Effect of CMC_ws_ Size Agent on Yarn Surface Structure

Considering the structural composition of yarn, it can be divided into three primary zones:

- An external layer composed of loosely oriented fibers with varying spacing relative to the yarn axis. This outermost region is inherently disordered and serves as the primary penetration zone for sizing agents in sized yarns.
- A subjacent layer of fibers, more directionally aligned, located at the interface between the disturbance zone and the cohesive core. This intermediate region contributes to both cohesion and disturbance functions.
- A core region, or undisturbed body of the yarn, characterized by tightly packed and well-aligned fibers that maintain the structural integrity of the yarn.

**Figure 3:**
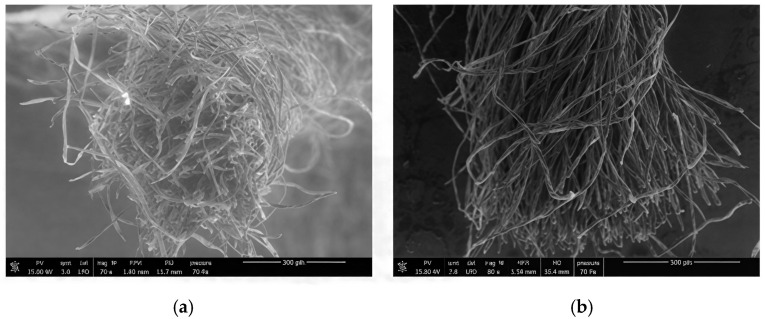

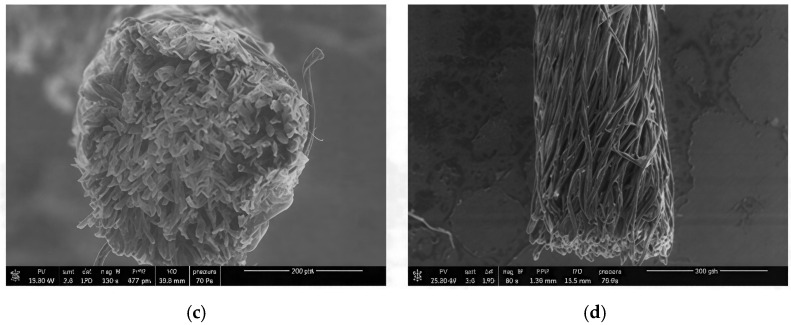
SEM images of unsized (**a**,**b**) and CMC_ws_-sized 100% cotton yarns (**c**,**d**).

The SEM micrographs in Figure 3 clearly demonstrate the effect of CMC_ws_ sizing on the surface morphology and hairiness of cotton warp yarns. The unsized yarn exhibits a high degree of hairiness, characterized by numerous protruding and loosely bound fibers extending from the yarn surface, which can increase friction, abrasion, and the likelihood of yarn breakage during weaving. In contrast, the CMC_ws_-sized yarn presents a markedly smoother and more compact surface, with most surface fibers firmly bound to the yarn body and aligned along the yarn axis. The pronounced reduction in protruding fibers indicates a significant decrease in yarn hairiness, attributed to the formation of a continuous and cohesive CMC_ws_ sizing film.

Due to the imperfect organization of fibrous elements, yarns inherently exhibit surface and structural defects, such as fiber ends, hooks, loops, gimlets, and neps. When subjected to mechanical stress, such as abrasion, these defects become more pronounced. Deformation leads to disruption of the sizing layer, fiber incoherence, and increased surface disturbance. The more intense the mechanical stress, the greater the disorganization of the fiber arrangement, ultimately resulting in the breakdown of the cohesion function and a corresponding increase in disturbance. This surface disturbance is quantitatively measured using a pilosimeter, which assesses yarn hairiness.

**Figure 4:**
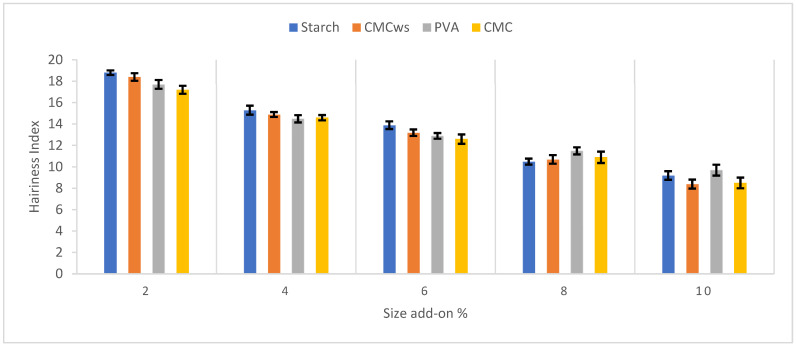
Hairiness index at different size add-ons.

As illustrated in Figure 4, yarn hairiness significantly decreases following sizing, regardless of the sizing material used (starch, PVA, CMC, or wheat-straw-derived CMC_ws_). This reduction in hairiness enhances the physical performance of the yarn, particularly under weaving conditions. However, with prolonged mechanical stress, the disturbance function increases due to gradual degradation of the sizing film. Nonetheless, maintaining the smoothness of the yarn surface and preserving the cohesive interface of the external fiber layer is essential for sustaining yarn performance during the weaving process.

For CMC_ws_-sized warp yarn, hairiness was reduced from 18.4 to 8.4, representing an approximate 54.35% reduction compared to the initial value at 2% size add-on. When comparing all types of samples, the greatest effect of “Size Add-on” on yarn hairiness was observed in the post-sizing values: 51.06% reduction for Starch, 54.34% for CMC_ws_, 45.19% for PVA, and 50.58% for CMC. The overall percentage reductions in yarn hairiness between 2% and 10% size add-on levels are summarized in Table 9, where the rankings of the agents are also identified. Different size add-ons were achieved by varying pickup percentage through adjustments in squeezing pressure. Among the tested agents, the low-cost CMC_ws_ showed a consistently higher reduction rate in yarn hairiness when compared to the commonly used sizing agents. Overall, CMC_ws_ proved to be an effective and economical sizing agent with strong performance (Figure 4).

Table 10 presents linear regression results obtained using Minitab^®^ 22 at a 95% confidence level. The analysis indicates that CMC_ws_ exhibits the strongest and most reliable performance among the evaluated sizing agents. The CMC_ws_ model shows a very high coefficient of determination (R^2^ = 98.95%) with a closely corresponding adjusted R^2^ (98.59%), demonstrating excellent explanatory power and model stability. In addition, the high F-value (281.56) and highly significant *p*-value (*p* < 0.001) confirm the strong influence of the studied factor on the response variable.

Compared with CMC, starch, and PVA, CMC_ws_ achieved the highest R^2^ and F-value, indicating a more consistent and predictable effect on yarn performance. These results suggest that CMC_ws_ forms an effective and uniform sizing film, resulting in reliable enhancement of yarn properties. Consequently, CMC_ws_ can be regarded as a highly effective sizing agent with excellent reproducibility.

### 3.5. Abrasion Properties of CMC_ws_-Sized Warp Yarns

The surface properties of woven fabrics directly reflect the performance of the yarns from which they are made. In this study, fabrics were produced from sized warp yarns, and their abrasion resistance behavior was evaluated. Therefore, fabric-level measurements serve as quantitative indicators of warp yarn durability and protection, providing a realistic assessment of yarn behavior under actual weaving conditions [29].

The difference in the number of abrasion cycles to failure between fabrics containing unsized yarns and those containing sized yarns is substantial, indicating that fabrics made from unsized yarns contain regions that are insufficiently protected and therefore mechanically weaker. These vulnerable regions are particularly susceptible to damage as the yarn passes through weaving elements such as drop wires, heald frames, and the reed. The relatively low number of abrasion cycles required to break unsized yarns clearly demonstrates that sizing treatment significantly enhances abrasion resistance.

Sized yarns require a greater number of abrasion strokes before the development of hairiness compared with unsized yarns. In unsized yarns, fibers are loosely arranged, whereas in sized yarns the fibers are bound together by the sizing agent. Fiber loosening begins only after the sizing film is disrupted by the repeated flexing action of the abrasion pins. As illustrated in Table 11, at low size add-on levels, the sizing skeleton breaks down rapidly after a limited number of abrasion strokes, resulting in lower abrasion resistance. In contrast, higher size add-on levels delay the breakdown of the sizing film, leading to improved abrasion resistance.

The apparent viscosity of CMC_ws_ solutions (903.03 cP) critically influences the properties of sizing films on cotton yarns. Higher viscosity, associated with increased polymer concentration, molecular weight, and DS (1.23), promotes greater size pick-up, forming thicker and more cohesive films that enhance mechanical protection, reduce fiber hairiness, and minimize breakage. Excessively high viscosity or DS, however, can produce stiffer films with reduced flexibility, potentially increasing brittleness [30].

Abrasion resistance increased progressively with increasing size add-on for all sizing agents, reflecting enhanced surface protection and improved fiber cohesion due to greater size deposition. Among the evaluated agents, PVA exhibited the highest abrasion resistance gain (37.50%), followed closely by CMC_ws_ (37.14%), indicating their superior film-forming properties and strong adhesion to the yarn surface. CMC showed a moderate improvement (31.20%), while starch produced the lowest gain (28.33%), suggesting comparatively weaker resistance to surface wear.

The CV% values (12.24–13.35%) indicate moderate variability and confirm acceptable experimental consistency. Notably, CMC_ws_ achieved abrasion resistance gains comparable to PVA while maintaining reasonable variability, highlighting its potential as an effective alternative sizing agent.

The regression results in Table 12, confirm that CMC_ws_ is an effective sizing agent, as evidenced by the high coefficient of determination (R^2^ = 96.61%) and statistically significant model (*p* < 0.001). The strong F-value indicates a pronounced effect of the studied factor on the response variable, while the close agreement between R^2^ and adjusted R^2^ reflects good model stability and reliable experimental behavior. Moreover, the consistently high performance of CMC_ws_ in improving mechanical properties, combined with acceptable variability, demonstrates its ability to form a uniform and durable sizing film. These findings support the suitability of CMC_ws_ as a reliable and efficient sizing agent, offering performance comparable to conventional agents.

### 3.6. Desizing Efficiency of CMC_ws_-Sized Warp Yarns

**Figure 5:**
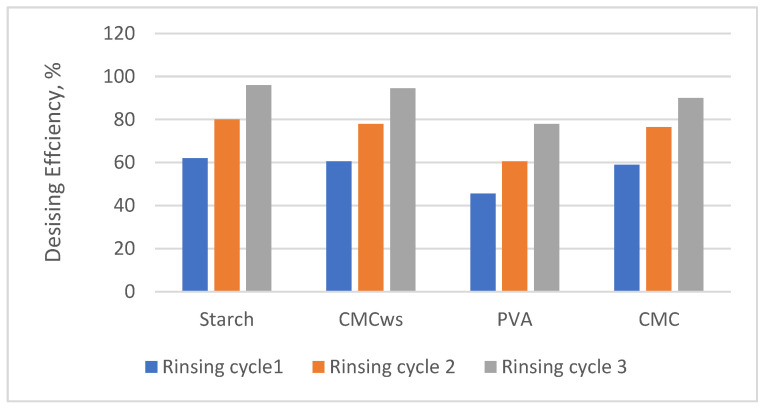
Desizing Efficiency of different sizing agents.

After weaving, the removal of sizing agents from fabrics is essential to ensure optimal dyeing and finishing quality. Therefore, good desizability is a critical property for textile sizing materials. The desizing efficiencies of sized cotton fabrics with different size recipes were shown in Figure 5. It could be seen clearly that the desizing efficiency was important, no matter what the used size recipe was employed. It is well established that the desizing efficiency of sizing agents is influenced by the viscosity of the size solution, the moisture regain of the size film, and the water solubility of the film. Increasing DS generally improves desizing efficiency due to enhanced solubility, but excessive DS can slightly compromise film mechanical integrity, requiring careful optimization for practical textile processing [17,27]. Undoubtedly, the hydrophilic character of the CMC_ws_ sizing agent accelerates the diffusion of chemicals into size material, which promotes the degradation and the removal of the size from the sized fabric. These data prove that the desizing efficiency of CMC_ws_-sized warp yarn exceeds 94%, indicating that CMCws-based sizes exhibit excellent ease of desizing.

Several studies have reported the use of various waxes for the development of super-hydrophobic surfaces and coatings [20]. In the context of CMC_ws_-based sizing, incorporating hydrophobic additives can create diffusion barriers, potentially affecting solution penetration, while the inherent hydrophilic nature of cotton fibers and CMC_ws_ films is largely maintained. This aligns with our desizing results, which show that hot-water desizing efficiently removes the CMC_ws_ sizing layer and preserves sufficient wettability, ensuring that the fabrics remain ready for subsequent treatments such as dyeing or finishing.

As shown in Table 13, the BOD_5_/COD ratio of the CMC_ws_ sizing agent reached approximately 0.50, indicating excellent biodegradability and effective microbial degradation. The efficient desizing behavior combined with the favorable degradation characteristics of the green CMC_ws_ developed in this study highlights its strong potential as a sustainable and environmentally friendly textile sizing agent. Nevertheless, while the reported BOD_5_/COD ratio confirms that CMC_ws_ is readily biodegradable under controlled laboratory conditions, its behavior in real wastewater systems may be influenced by variations in organic load, microbial community structure and activity, as well as environmental parameters such as pH and temperature [31,32].

In contrast, the PVA sizing agent exhibited a much lower BOD_5_/COD ratio of approximately 0.20, confirming its limited biodegradability and persistence in aqueous environments. Although conventional polyvinyl alcohol (PVA) is characterized by a relatively low biodegradation rate, leading to poor biodegradability indicators, recent studies have demonstrated that modified, blended, or partially hydrolyzed PVA formulations can significantly improve biodegradability, while simultaneously enhancing water solubility and microbial degradation [33].

### 3.7. Evaluation of Cost-Effectiveness

**Figure 6:**
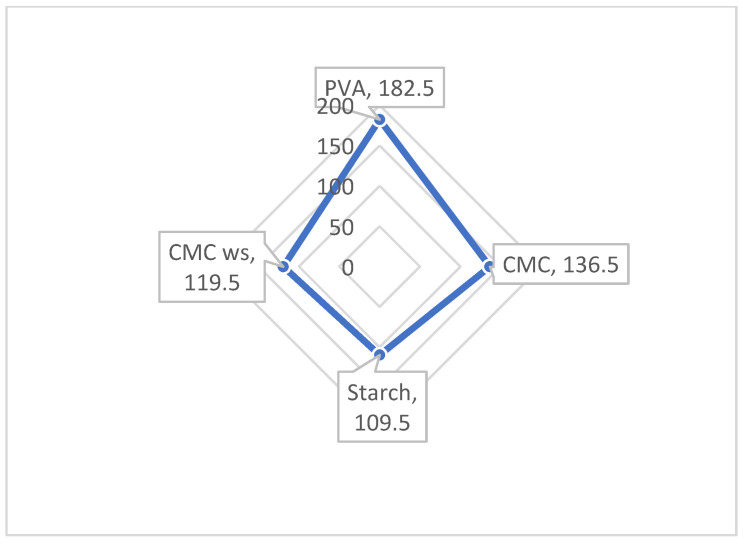
Sizing chemical cost analysis (500 L solution).

A comparative cost analysis of various sizing agents for 500 L solution volumes is presented in Figure 6. This evaluation is crucial for assessing the industrial viability of carboxymethyl cellulose derived from wheat straw (CMC_ws_) as a sustainable alternative.

As shown in Table 14, the cost price of each size formulation was determined by summing the individual costs of all components used in the recipe, including the primary sizing agents and auxiliary additives. The calculation considered the quantity of each compound in the formulation, expressed in grams per liter (g/L), and its corresponding market price per kilogram. For each ingredient, the total cost was obtained by multiplying its mass (converted to kilograms for the total batch volume) by its unit cost.

The results clearly demonstrate the economic advantage of CMC_ws_-based sizing formulations. Specifically, the cost of a conventional polyvinyl alcohol (PVA)-based sizing agent for 500 L is approximately US$182.5, whereas the CMC_ws_-based formulation incurs a lower cost of US$119.5. This indicates that the use of CMC_ws_ can lead to a substantial reduction in material costs. Therefore, CMC_ws_ emerges as a cost-effective and competitive alternative to traditional sizing agents widely used in the industry.

## 4. Conclusions

This study investigated the use of carboxymethyl cellulose (CMC_ws_) derived from Saudi wheat straw as a low-cost, biodegradable alternative to conventional cellulose-based sizing agents for cotton warp yarns. The research highlighted both the environmental and economic advantages of utilizing wheat straw-derived CMC_ws_ and evaluated its effectiveness in textile applications. A comparative analysis was carried out to assess the weavability of cotton warps sized with wheat straw-derived CMC_ws_ against traditional sizing agents, including maize starch, polyvinyl alcohol (PVA), and industrial-grade commercial CMC.

The evaluation focused on key yarn performance indicators such as breaking force, elongation at break, abrasion resistance, and overall weaving efficiency. The adhesive power of the CMC_ws_ sizing agent was identified as a critical factor in its suitability, as it directly influences the yarn’s abrasion resistance by enhancing fiber cohesion.

The results clearly demonstrated that the CMC_ws_ sizing agent falls within an acceptable performance range for warp sizing. As a sustainable and efficient alternative, CMC_ws_ derived from agricultural waste present a promising solution for the textile weaving industry. This approach supports the move towards greener production practices, making CMC_ws_ a strong candidate for meeting the current demands of cotton yarn sizing in an environmentally responsible and cost-effective manner.

## Figures and Tables

**Table 1:** Composition and preparation parameters of size formulations.

Parameter	Value
Size concentration, %	0.5–8
Plasticizer concentration, %	8
Lubricant concentration, %	7
Viscosity, cP	60–200
Mixing Temperature, °C	90
pH	7.0
Duration, h	2

**Table 2:** Biodegradation performance testing parameters and conditions.

Parameter	Description/Condition
Test Temperature	20 ± 1 °C
pH	7.0 ± 0.2
Atmosphere	Aerobic
Incubation Period (BOD_5_)	5 days
Inoculum Source	Activated sludge (municipal wastewater treatment plant)
Replicates	3 per sizing agent
Standards Used	COD—GB 11914-89; BOD_5_—HJ 505-2009
Biodegradability Indicator	BOD_5_/COD ratio
Interpretation Criteria	>0.4: readily biodegradable; <0.3: poorly biodegradable

**Table 3:** Chemical composition (*w*/*w* %) of Saudi durum wheat straw in comparison with previously reported values.

Composition	Saudi Durum Wheat Straw	Khiari et al. [28]
Solvent extractives	7.2 ± 1	4.6–9.2
Hot water solubility	12 ± 0.8	14
1% NaOH solubility	41 ± 1	41–42.8
Ash	4 ± 0.5	4–9
Klason lignin	14 ± 0.8	11–21
α-Cellulose	41 ± 1	33–45.5

**Table 4:** Cotton warp yarns’ breaking strength at different size concentrations.

Size Concentration, %	Size Agent			
	Starch	CMC_ws_	PVA	CMC
0.5	17.2 ± 0.22	17.5 ± 0.35	18.5 ± 0.41	18.1 ± 0.37
2	18 ± 0.42	19.1 ± 0.23	21 ± 0.34	20.03 ± 0.25
4	19 ± 0.36	20.5 ± 0.30	23 ± 0.27	22 ± 0.44
6	20 ± 0.28	21.9 ± 0.40	24 ± 0.33	23.1 ± 0.53
8	21 ± 0.40	22.5 ± 0.42	25 ± 0.51	24 ± 0.50
CV, %	10.23	9.36	11.04	11.57
Breaking strength gain, %	22.09	28.57	35.13	32.59

**Table 5:** Regression analysis results of breaking strength (cN/Tex) of sized yarns versus concentration.

Source	SS	MS	F-Value	*p*-Value	R^2^	Ra^2^	Remarks
CMC_ws_ Regression	16.1779	16.1779	89.53	0.003	96.76%	95.68%	Significant
CMC Regression	22.6541	22.6541	57.76	0.005	95.06%	93.42%	Significant
Starch Regression	9.23090	9.23090	94.61	0.000	99.99%	99.98%	Significant
PVA Regression	25.0279	25.0279	42.37	0.007	93.39%	91.18%	Significant

**Table 6:** Tensile properties of the yarn.

Yarn Property	Unsized	Sized							
		Low Pressure 220 N/m				High Pressure 330 N/m			
		Starch	CMC_ws_	PVA	CMC	Starch	CMC_ws_	PVA	CMC
Breaking Strength, cN/tex	17.1	18.09 ± 0.25	17.54 ± 0.23	20.67 ± 0.24	18.23 ± 0.21	18.65 ± 0.27	17.77 ± 0.19	21.19 ± 0.22	19.15 ± 0.19
Breaking Strength CV, %	13.53	14.32	13.23	12.46	12.81	18.25	21.24	17.28	23.62
Breaking Elongation, %	5.6 ± 0.15	4.4 ± 0.14	3.9 ± 0.13	4.2 ± 0.15	3.7 ± 0.14	4.2 ± 0.17	3.6 ± 0.15	4 ± 0.17	3.5 ± 0.18
Breaking Elongation CV, %	18.13	15.67	17.42	20.48	18.15	12.02	16.05	16.16	15.41

**Table 7:** Packing density of CMC_ws_-sized Yarn.

Yarn Packing Property	Unsized Yarns	Sized Yarns							
		Low Pressure 220 N/m			High Pressure 330 N/m				
		Starch	CMC_ws_	PVA	CMC	Starch	CMC_ws_	PVA	CMC
Packing Density	0.548	0.593	0.629	0.651	0.637	0.618	0.651	0.663	0.658
Packing Density CV, %	18.21	12.32	13.87	11.27	15.02	14.54	13.73	16.23	15.36

**Table 8:** CMC_ws_ size films’ cohesion force compared to other sizing materials.

	Size Material			
	Starch	CMC	PVA	CMC_ws_
Thickness, mm	0.01	0.01	0.01	0.01
Cohesion, MPa	8.64 ± 0.31	11.48 ± 0.37	13.89 ± 0.23	10.24 ± 0.43
Cohesion, MPa CV, %	16.32	12.98	14.57	11.23

**Table 9:** Hairiness Index of different agents sized yarns.

Size Add-on, %	Size Agent			
	Starch	CMC_ws_	PVA	CMC
2	18.8 ± 0.12	18.4 ± 0.15	17.7 ± 0.21	17.2 ± 0.37
4	15.3 ± 0.14	14.9 ± 0.13	14.5 ± 0.24	14.2 ± 0.25
6	13.9 ± 0.16	13.2 ± 0.17	12.9 ± 0.27	12.6 ± 0.44
8	10.5 ± 0.22	10.7 ± 0.25	11.5 ± 0.13	10.9 ± 0.53
10	9.2 ± 0.20	8.4 ± 0.31	9.7 ± 0.19	8.5 ± 0.50
CV, %	12.03	11.06	11.42	10.17
Hairiness Reduction, %	51.06%	54.34%	45.19%	50.58%

**Table 10:** Regression Analysis of Yarn Hairiness Index as a Function of Size Add-on for Different Sizing Agents.

Source	SS	MS	F-Value	*p*-Value	R^2^	Ra^2^	Remarks
CMC_ws_ Regression	58.564	58.564	281.56	0.000	98.95%	98.59%	Significant
CMC Regression	42.849	42.849	238.49	0.001	98.76%	98.34%	Significant
Starch Regression	57.600	57.600	125.95	0.002	97.67%	96.90%	Significant
PVA Regression	36.100	36.100	102.95	0.002	97.17%	96.22%	Significant

**Table 11:** Abrasion resistance of different agents sized yarns.

Size Add-on, %	Size Agent			
	Starch	CMC_ws_	PVA	CMC
2	600 ± 8.26	700 ± 7.15	800 ± 8.23	750 ± 9.31
4	660 ± 10.14	780 ± 8.23	900 ± 8.54	800 ± 9.25
6	680 ± 10.16	840 ± 9.37	942 ± 10.25	850 ± 10.14
8	710 ± 8.12	900 ± 10.15	1000 ± 9.58	922 ± 10.51
10	770 ± 7.42	960 ± 10.51	1100 ± 10.09	984 ± 90.63
CV, %	12.54	13.26	12.24	13.35
Abrasion resistance gain, %	28.33%	37.14%	37.50%	31.20%

**Table 12:** Regression analysis results of abrasion resistance of different-sized yarns versus size add-on.

Source	SS	MS	F-Value	*p*-Value	R^2^	Ra^2^	Remarks
CMC_ws_ Regression	40,960	40,960	768.00	0.000	96.61%	99.48%	Significant
CMC Regression	34,810	34,810	514.94	0.000	99.42%	99.23%	Significant
Starch Regression	15,210	15,210	89.47	0.003	96.76%	95.67%	Significant
PVA Regression	49,000	49,000	139.84	0.001	97.98%	97.20%	Significant

**Table 13:** BOD/COD of the desized wastewater.

Type of Size	BOD_5_/COD
Starch	0.55
CMC_ws_	0.54
PVA	0.20
CMC	0.50

**Table 14:** Cost estimation of sizing agents (at 6%) per 500 L solution (2025 prices).

Sizing Agent/ Component	Quantity per 500 L	Unit Price (USD)	Total Cost (USD)	Notes
PVA	30 kg	5.41	162.5	Local supplier (SITEX, Tunisia)
CMC	30 kg	3.88	116.5	Local supplier (SITEX, Tunisia)
Starch	30 kg	2.98	89.5	Local supplier (SITEX, Tunisia)
CMC_ws_ (synthesized)	30 kg	–	119.5	
Wheat straw	40 kg	2.48	99.5	
NaOH	5 kg	1.5	7.5	Sigma-Aldrich
MCA	12.5 kg	5	62.5	Sigma-Aldrich
Ethanol	15 kg	2	30.0	Sigma-Aldrich
Plasticizer/Lubricant	2 kg	10.0	20.0	Sigma-Aldrich
Total CMC_ws_	–	–	119.5	Sum of all components

## Data Availability

The original contributions presented in this study are included in the article. Further inquiries can be directed to the corresponding author.

## References

[1] Drean J.-Y., Decrette M., Kyosev Y., Boussu F. (2022). Weaving Preparation. Advanced Weaving Technology.

[2] Maatoug S., Ladhari N., Sakli F. (2007). Evaluation of the weavability of sized cotton warps. Autex Res. J..

[3] Kovačević S., Penava Ž. (2004). Impact of sizing on physico-mechanical properties of yarn. Fibres Text. East. Eur..

[4] Maatoug S., Ladhari N., Sakli F. (2007). Fatigue behavior of sized cotton warps. J. Appl. Sci..

[5] Walker R.P., Perkins W.S. (1985). Effect of sizing wax on tensile properties, abrasion resistance, and weaving performance of polyester/cotton yarn sized with polyvinyl alcohol. Text. Res. J..

[6] Muhammad N.A., Muhammad A.S., Khan S., Kaffayatullah K. (2022). Influence of Fineness of Wheat Straw Ash on Autogenous Shrinkage and Mechanical Properties of Green Concrete. Crystals.

[7] Sun S., Yu H., Williams T., Hicks R.F., Qiu Y. (2013). Eco-friendly sizing technology of cotton yarns with He/O2 atmospheric pressure plasma treatment and green sizing recipes. Text. Res. J..

[8] Kadam K.L., Forrest L.H., Jacobson W.A. (2000). Rice straw as a lignocellulosic resource: Collection, processing, transportation, and environmental aspects. Biomass Bioenergy.

[9] Laribi N., Maatoug S., Jebali Z., Zouari R., Majdoub H., Cheikhrouhou M. (2020). Low-cost carboxymethyl holocellulose and carboxymethyl cellulose from wheat straw. Cellul. Chem. Technol..

[10] Wise L.E., Murphy M., D’Addieco A.A. (1946). A chlorite holocellulose, its fractionation and bearing on summative wood analysis and studies on the hemicelluloses. Paper Trade J..

[11] Mansouri S., Khiari R., Bettaieb F., El-Gendy A., Mhenni M.F.J. (2015). Synthesis and Characterization of Carboxymethyl Cellulose from Tunisian Vine Stem: Study of Water Absorption and Retention Capacities. Polym. Environ..

[12] El-Sheikh M.A. (2010). Carboxymethylation of maize starch at mild conditions. Carbohydr. Polym..

[13] Hebeish A., Guthrie T. (2012). The Chemistry and Technology of Cellulosic Copolymers.

[14] Zhou Z., Xia K., Liu T., Guo Liu H., Zhang X. (2022). Preparation of carboxymethyl cellulose nanofibers and their application in warp size of textile. Int. J. Biol. Macromol..

[15] (1990). Textiles—Yarns—Determination of the Abrasion Resistance of Sized Warp Yarns.

[16] Pan X., Sano Y. (2005). Fractionation of wheat straw by atmospheric acetic acid process. Bioresour. Technol..

[17] Lopez C.G., Richtering W. (2021). Oscillatory rheology of carboxymethyl cellulose gels: Influence of concentration and pH. Carbohydr. Polym..

[18] Fernando E.A.S.K., Jayawardana T.S.S. (2015). Development of Mathematical Model to Select Best Technological Parameters in Sizing. J. Multidiscip. Eng. Sci. Technol..

[19] Gandhi K.L. (2016). Yarn Preparation for Weaving: Sizing. Woven Textiles.

[20] Rahmouni A., Maatoug S., Ladhari N. (2024). The effect of size concentration, lubricant and plasticizer agents contents on cold sizing process performance of cotton warp yarns: Modelling and optimization studies. J. Text. Inst..

[21] Behera B., Gupta R. (2008). Comparative analysis of desizability and retrogradation behavior of various sizing materials. J. Appl. Polym. Sci..

[22] Djordjevic S., Kovacevic S., Nikolic L., Miljkovic M., Djordjevic D. (2014). Cotton yarn sizing by acrylamide grafted starch copolymer. J. Nat. Fibers.

[23] Farag R., Elmogahzy Y. (2009). 3—Tensile properties of cotton fibers. Handbook of Tensile Properties of Textile and Technical Fibres.

[24] Ayele M., Genene Abay A. (2023). Analyzing the Effect of Various Sizing Machine Settings on Abrasion Resistance and Size Pick-Up of Polyester/Cotton Blend Sized Yarn Using Box-Behnken Design. J. Nat. Fibers.

[25] Devare M.D., Turukmane R.N., Gulhane S.S., Patil L.M. (2016). Effect of Yarn Stretch in Sizing on Loom Performance. Int. J. Text. Eng. Process..

[26] Djordjevic S., Kovacevic S., Djordjevic D., Konstantinovic S. (2019). Sizing process of cotton yarn by size from a copolymer of methacrylic acid and hydrolyzed potato starch. Text. Res. J..

[27] Yang M., Xu H., Hou X., Zhang J., Yang Y. (2017). Biodegradable sizing agents from soy protein via controlled hydrolysis and dis-entanglement for remediation of textile effluents. J. Environ. Manag..

[28] Khiari R., Mhenni M.F., Belgacem M.N., Mauret E. (2010). Chemical composition and pulping of date palm rachis and *Posidonia oceanica*—A comparison with other wood and non-wood fibre sources. Bioresour. Technol..

[29] Hermann D., Ramkumar S.S., Seshaiyer P., Parameswaran S. (2004). Frictional Study of Woven Fabrics: The Relationship between the Friction and Velocity of Testing. J. Appl. Polym. Sci..

[30] Esmat B., Bibi S., Soliman M.M., Thabet H.K., Wattoo M.A., Nazir M.A., El-Bahy Z.M., Rehman A. (2026). Carboxymethyl cellulose: Structural modifications and their impact on barrier and mechanical properties for sustainable food packaging. J. Stored Prod. Res..

[31] Gupta S., Ivvala J., Grewal H.S. (2021). Development of natural wax based durable superhydrophobic coatings. Ind. Crops Prod..

[32] Zhang X., Baek N.W., Xu J., Yuan J., Fan X. (2022). Differences in the desizability of starches and the mechanism of inhibiting desizing. Text. Res. J..

[33] Elgharbawy A.S., El Demerdash A.M., Sadik W.A., Kasaby M.A., Lotfy A.H., Osman A.I. (2024). Enhancing the Biodegradability, Water Solubility, and Thermal Properties of Polyvinyl Alcohol through Natural Polymer Blending: An Approach toward Sustainable Polymer Applications. Polymers.

